# Meeting Report: Vaccine Stability Considerations to Enable Rapid Development and Deployment

**DOI:** 10.1186/s41120-021-00042-1

**Published:** 2021-12-01

**Authors:** Mark Alasandro, Dilip Choudhury, Kim Huynh-Ba, Jianmei Kochling, Christopher Latoz, Laure Larkin, Lori McCaig, Nanda Subbarao, Yan Wu, Yajie Zhang

**Affiliations:** 1 Adamis Pharmaceuticals, San Diego, CA USA; 2 Cary, NC, USA; 3 Pharmalytik, LLC, Newark, DE USA; 4 Sanofi, Boston, MA USA; 5 Hollister Incorporated, Libertyville, IL USA; 6 Johnson and Johnson, Raritan, NJ USA; 7 Seagen Inc., Bothell, WA USA; 8 Biologics Consulting, Alexandria, VA USA; 9 Merck & Co, Inc., Kenilworth, NJ USA; 10 Regeneron Pharmaceuticals, Tarrytown, NY USA

**Keywords:** Vaccine Development, COVID-19, Stability, Rapid Development, Rapid Deployment, Last-Mile, Shipping Study, Freeze-Thaw Study, In-Use Study, Pharmaceutical Supply Chain, Drug Product Transport, Stability Modeling, Platform Technologies

## Abstract

The Stability Community of the American Association of Pharmaceutical Scientists (AAPS) held a virtual workshop on “Vaccine Stability Considerations to Enable Rapid Development and Deployment”, on March 24-25, 2021. The workshop included distinguished speakers and panelists from across the industry, academia, regulatory agencies, as well as health care leaders. This paper presents a review of the topics covered. Specifically the challenges in accelerating vaccine development and analytical characterization techniques to establish shelf-life were covered. Additionally, vaccine stability modeling using prior knowledge stability models and advanced kinetic analysis played a key in the EUA approaches discussed during the workshop. Finally, the role of stability studies in addressing the challenges of vaccine distribution and deployment during the pandemic were a focus of presentations and panel discussions.

Although the workshop did not have any presentation topics directly dedicated to the mRNA vaccines, the techniques discussed are generally applicable. The mRNA vaccine developers were represented in the panel discussions, where experts involved in the EUA approval/deployment stages for this vaccine type could discuss the challenges as applied to their vaccines.

## Introduction

Vaccines have always been a focus of worldwide public health organizations. This global scrutiny has sharpened significantly during the current COVID-19 pandemic. The urgent need for a safe and effective vaccine has pushed science and technologies to their limits in order to accelerate vaccine development and deployment. Stability studies must be designed into the development of new vaccines, starting with the formulation selection. This integrated stability approach must carry through the subsequent processing, packaging, storage, and distribution phases. The ever-expanding range of different vaccine modalities, combined with the need to maintain vaccine quality through a complex global supply chain, presents stability testing design challenges. Fortunately, the analytical tools and stability modelling and data leveraging strategies are advancing in parallel. Stability scientists must be aware of current best practices to ensure stability programs are optimized to support urgent product introductions without sacrificing quality, safety, and efficacy.

The Stability Community of the American Association of Pharmaceutical Scientists (AAPS) conducted a virtual workshop from March 24-25, 2021. The purpose of the workshop was to facilitate the discussion about the latest developments in vaccine stability strategies with a particular emphasis on COVID-19 vaccines. To address the pandemic, a highly effective vaccine(s) had to be developed quickly. To be effective, any vaccine had to be deployed with effective supply chain controls and in-use (last mile) vaccination program. Both the general stability of the vaccine and its rapid deployment were critical. The need for compressed timelines rendered the traditional ICH Q5C and real-time expiry dating study approach insufficient for the urgency of the vaccine need. The storage conditions and distribution challenges of the new modality COVID-19 vaccines, such as mRNA and Adenovirus types, also place increased focus on short-term auxiliary studies to address excursions during distribution and local storage at global injection sites. This meeting was intended to act as a forum to discuss different strategies to confirm the stability of vaccines for global emergency vaccination programs.

### Part I – Vaccine Formulation Challenges in a Pandemic Setting

The first session of the workshop was focused on recent trends in the accelerated development of vaccines. Dr. Lisa Kueltzo from NIH presented a drug development process for meeting the urgent needs during the pandemic, starting from multiple different therapeutic modalities. Regardless of different approaches, most commenced with a target product profile (TPP). However, the pandemic response TPP may be quite different from a traditional program TPP, because speed became a critical factor. “Results of numerous research efforts in the preceding decade have established platform technologies to accelerate the vaccine development for SARS-COV-2. The key to success in developing vaccines as a fast response to the global needs is out of the box thinking to modify both traditional development and stability strategy and proactively generating vaccine and drug development programs for perhaps the inevitable next global health crisis”, Dr. Kueltzo stated.

The COVID-19 vaccine development success was supported by government funding commitment, public engagement, and rapid response from regulatory agencies. However, the selection of strengths can also become constraints, as the vaccine development timeline was compressed from years to months, resulting in a supply chain disruption and a high demand for personnel resources. The tolerance for human error can impact on highly compressed pandemic vaccine development timelines. Moreover, developing stable vaccines that can be stored in manageable storage conditions is challenging, yet it is a significant consideration to increase efficiency in a pandemic setting.

There are a variety of regulatory approval mechanisms that are used by health authorities, including emergency use authorization (EUA) or accelerated approval processes, to work together with sponsors to get vaccines to patients in record time. The focus during the pandemic caused a change in the emphasis from Cost to Speed. However, the TPP consideration remains the same with or without the pandemic. Rolling submission of clinical drug product stability data is also supported by the regulatory agencies, which allows active collaboration and facilitates decisions effectively. Direct communication with the clinical design team is very critical during a pandemic to ensure the stability testing is appropriate to support the storage and deployment of the product.

**Fig. 1:**
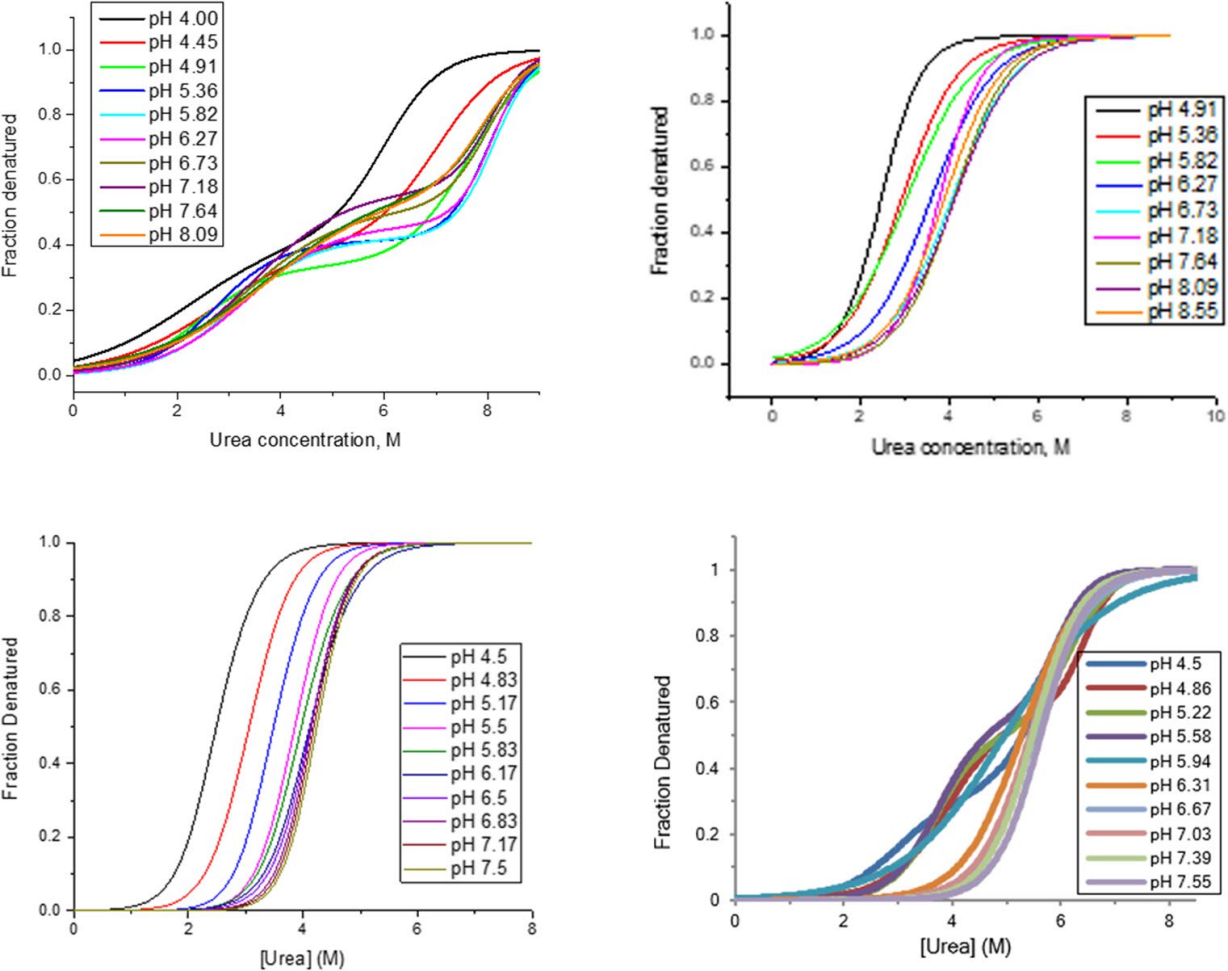
Leveraging Historical Data - Effect of pH on Viral Fusion Proteins (FusProtX). Excerpt from Kueltzo, L., Vaccine Development Challenges in a Pandemic Setting: Formulation and Stability Considerations, AAPS Vaccine Stability Workshop, March 24-25, 2021

Moreover, due to the compressed timeline, the ‘platform’ development process has been applied not only to vaccines but also to monoclonal antibodies and other therapeutics, of which prior knowledge was used to speed up the development. Dr. Kueltzo presented an example of how historical data can be leveraged to evaluate different vaccines. Figure 1 shows the isothermal chemical denaturation using urea, where pH was used to quickly decide on which pH to target the formulation. Similar behaviors are observed for various formulation factors and analytical procedures such as adjusting ionic strength, using size exclusion chromatography, high throughput fluorescence intrinsic or extrinsic differential scanning fluorimetry studies. Various quality attributes may need to be balanced by formulation parameters (e.g., higher conformational stability may come at the expense of increased chemical degradation). Therefore, platform technologies to evaluate new vaccines and streamline the process is increasingly used for new product development.

In addition, the availability of the materials and personnel may also impact the method used. As the pharmaceutical industry has been under tremendous pressure to accelerate its product development and identify an effective vaccine, increased communication with regulatory agencies is the key. Different types of development stability data to support the vaccine launch, included:

- designing early stability studies to represent GMP late-stage studies
- using multiple development lots
- leveraging toxicology data from aged lots
- representing container-closure packaging systems for clinical studies

The session continued as Dr. Michael Angelastro from the BARDA, a US Health Services organization, and Dr. Gayle Pulle from Health Canada joined Dr. Lisa Kueltzo from NIH for the panel discussion. Dr. Angelastro gave an overview of BARDA and their involvement in the vaccine development globally for diseases such as Ebola, Zika, some of the Influenza strains, and now COVID-19. He indicated that one of the challenges is the need to learn about supply chains. In most cases, there is not a clear understanding of supply chains such as availability of glass vials, needles, syringes, adjuvants, or bills of materials for different vaccines. The manufacturing choke points must be understood to minimize manufacturing delay.

Dr. Pulle covered the minimum stability data required for vaccine development. She emphasized the need for active communication between regulatory agencies of various jurisdictions during the vaccine approval process. Dr. Pulle also indicated that the platform development is quite useful in the acceleration of vaccine development, and it also helps to facilitate data review among reviewers. She noted that the available predictive tools have different success rates depending on vaccine type or modality, and the most available models are designed for one molecule rather than a broad category of different molecules. In addition, there can be a limitation of materials and resources to conduct these experiments.

Following the talks, the panel discussed the challenges of working with multi-dose presentations due to the lack of preservatives. A study may be needed about whether multi-punctures can lead to contaminations. It was noted that although new delivery systems may be beneficial for long-term vaccine lifecycle and use, complex delivery systems may cause delays in the development as well as deployment. Therefore, dose delivery was kept as simple as possible during the pandemic. To address the needs during a pandemic, companies should build capacity and redundancies in the supply chain, implement an optimized process, and make additional collaborative efforts to ensure a smooth transition.

Although facing many challenges from limited capacity and technical resources to supply chain, all panelists agreed that it is personally satisfying to contribute to the efforts in ensuring that quality vaccines are developed at the time of need. In January 2021, the Food and Drug Administration (FDA) also issued guidance on “COVID-19: Potency Assay Considerations for Monoclonal Antibodies and Other Therapeutic Proteins Targeting SARS-CoV-2 Infectivity,” to assist sponsors in the development of monoclonal antibodies (mAbs) and other therapeutic proteins for use as COVID-19 therapeutics. The goal of the guidance is to ensure that each lot is consistently produced with the potency assay needed to achieve clinical efficacy and such potency is maintained over the shelf-life of the product (Food and Drug Administration (FDA) Guidance for Industry, COVID-19, 2021).

Further discussion on different predictive tools can be found in Part III.

### Part II – Analytical Characterization to Establish Shelf-Life

Dr. Arjen Scholten, Janssen Vaccines and Prevention BV, Johnson & Johnson, presented the company’s stability and shelf-life approach to support the EUA submission for the COVID-19 vaccine. Though Janssen had previously used the same Adenovirus vector (AdVac® 26) platform technology for the development of the Ebola vaccine, developing the COVID-19 vaccine was not without challenges. Trying to reliably estimate shelf-life, which can be acceptable to regulatory agencies for EUA approval, was an enormous challenge given that the time between Phase 1 clinical trial and EUA submission was approximately 7 months, and there was very limited real-time stability data available for the COVID-19 vaccine at that time.

Dr. Scholten described how they built the initial shelf-life model for the COVID-19 vaccine product leveraging the AdVac® 26 platform data and prior knowledge of products based on this platform. More than 50 batches of drug substances manufactured utilizing AdVac® 26 and PER.C6® cell lines were used for building the model. Potency was monitored by infectivity (cell-based PCR). The initial specifications for drug product release were based on the manufacturing data for the Ebola process. Conservative allowances were built-in for losses during packaging, supply chain, and administration to the patient's needs. Worst case representative platform slope, along with additional uncertainty-related loss for shelf-life prediction, was applied. The strategy was to replace the platform data with COVID-19 specific data as they become available.

**Fig. 2:**
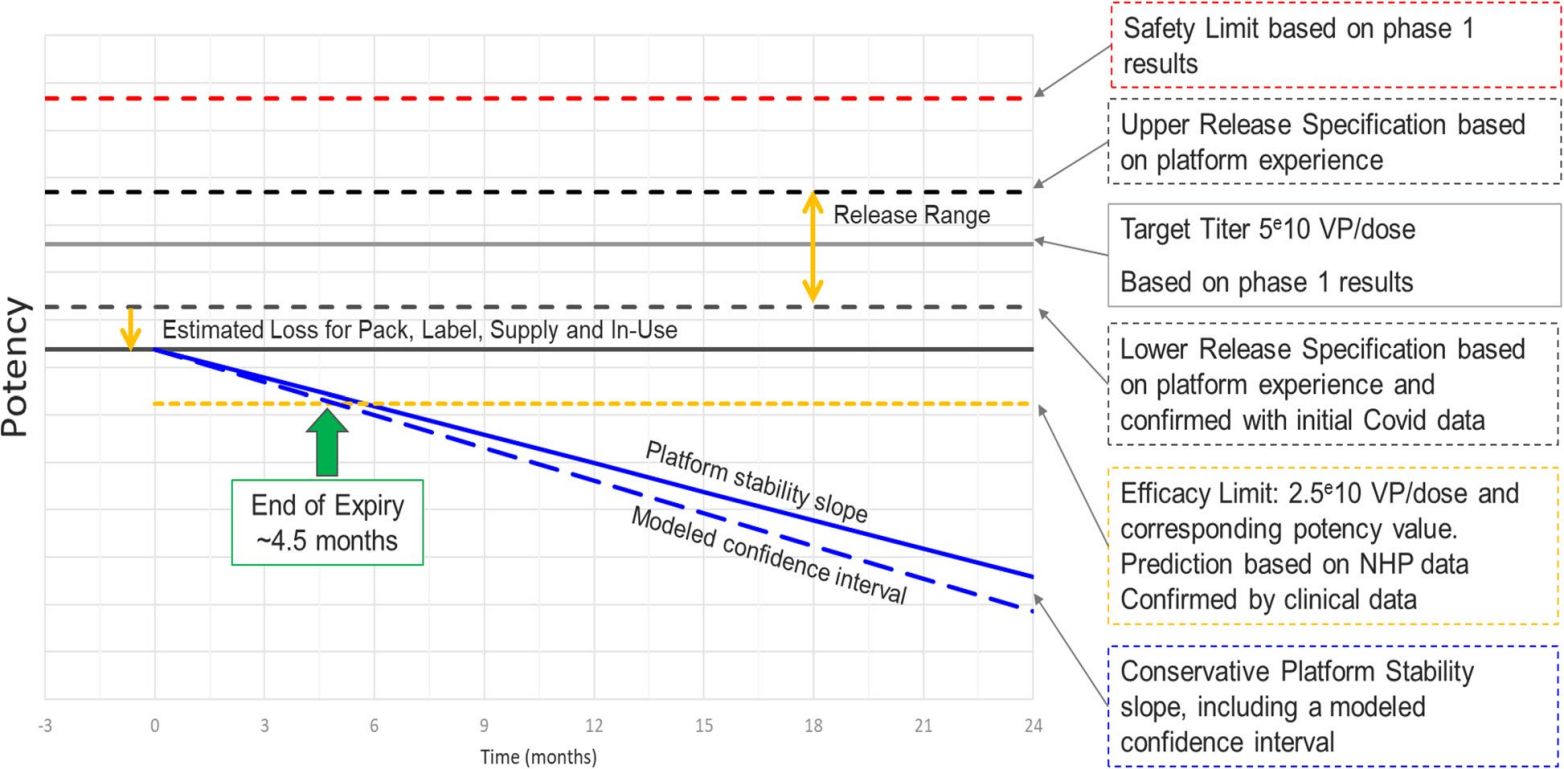
Janssen Shelf-life Model used for COVID-19 Vaccine. Excerpt from Scholten, A., Defining Shelf-Life for the COVID-19 Vaccine EUA, AAPS Vaccine Stability Workshop, March 24-25, 2021

For the shelf-life assignment, stability data of more than 50 batches of AdVac® platform-based drug substance was utilized. Figure 2 describes the stability model of the vaccine potency as a function of time. When stored at -85 to -40°C, no significant slope for potency was observed for up to 36 months. No significant slope for potency was also observed when stored at -25 to -15°C for up to 36 months. Based on these platform data, 24 months shelf-life was assigned at -25 to -15°C. A downward trend was observed upon storage at 2 – 8°C. Based on the slope of drug product stored in an inverted position (worst case), the slope of representative platform materials (n=23) and adding additional allowances for uncertainty, 3 months shelf-life was assigned at 2 – 8°C. The lower limit of the specification was initially set based on non-clinical data and confirmed by clinical efficacy data just prior to submission of EUA.

The panel also discussed additional approaches, including a suite of orthogonal analytical techniques such as SDS-PAGE, RP-HPLC, MALS, LC-MS, etc., and how they have been applied for characterization of the spike protein in protein-based vaccines. Like any therapeutic product, vaccine drug substances and drug products need to be fully characterized. For recombinant protein-based vaccines, the spike protein must be thoroughly characterized utilizing appropriate techniques for structure and conformation, potency, purity, impurities, exact molecular mass, and impact of formulation buffer on stability. Potency and purity methods must be stability-indicating and all methods must be specific and sensitive enough to detect any changes in the product at all stages of manufacturing and shelf-life.

These presentations in this section discussed the importance of thorough characterization of the vaccine candidate and the spike protein construct involved as appropriate. If a platform technology is used for the vaccine candidate development and manufacture, prior knowledge of platform-based stability data may be leveraged for shelf-life projection with scientific justification. Such projection is extremely helpful for accelerating vaccine development, and with appropriate scientific rationale, may be acceptable to regulatory authorities for EUA in a pandemic situation.

### Part III – Principles and Practices of Vaccine Stability Modeling

**Fig. 3:**
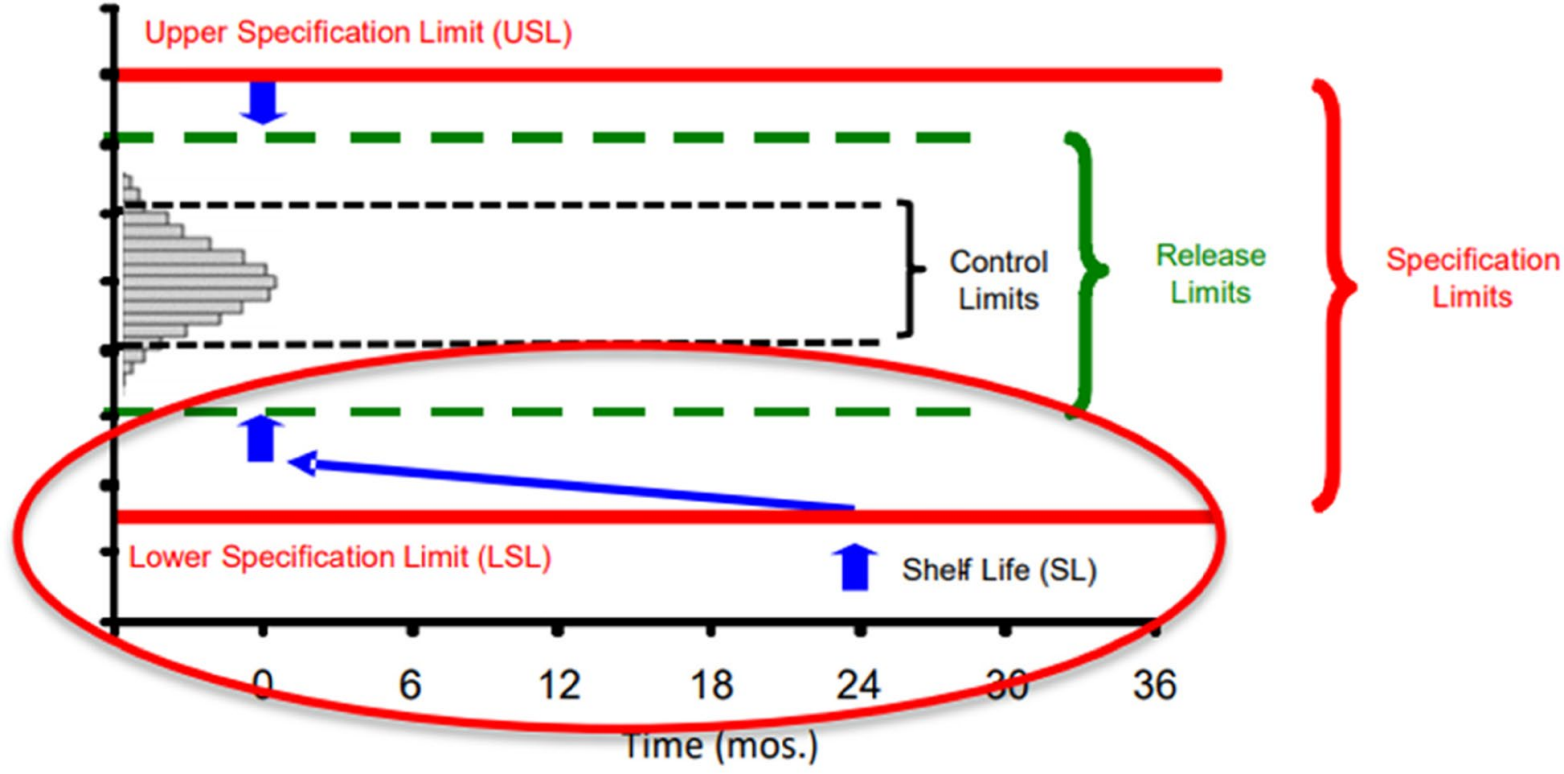
Vaccine Analytical Control Strategy. Excerpt from Schofield, T., Principles and Practices of Vaccine Stability Modeling. AAPS Vaccine Stability Workshop, March 24-25, 2021

Mr. Tim Schofield, CMC Sciences, presented a talk on the general principles and practices of vaccine stability modeling, highlighting the principles in the WHO Guidelines on Stability Evaluation of Vaccines (World Health Organization (WHO), 2006; World Health Organization (WHO), 2015) and illustrating their implementation. Due to an inherent instability, most vaccines typically require storage at refrigerated 2-8°C to frozen (-20 to -70°C) conditions. Using a vaccine stability model, the release limits are set to ensure the quality of vaccines throughout their designated shelf-life. Specific temperatures used in the stability modeling may include those required for shipping or at the end-user (the vaccination site for example) (World Health Organization (WHO), 2006; World Health Organization (WHO), 2015). Mr. Schofield contrasted such statistical modeling with less rigorous approaches or routine monitoring. The resulting vaccine statistical stability models result in less risk to the manufacturer and patients over the shelf-life than merely monitoring stability measurements against the vaccine specification limits (Schofield, 2009). Figure 3 shows the typical model for a vaccine analytical control strategy, where the lower and upper specification limits are scientifically or clinically justified, release limits are calculated to ensure quality is achieved at release and maintained throughout shelf-life, and control limits are designed to manage manufacturing consistency.

Mr. Schofield also presented statistical approaches for post-licensure stability modeling. Data can be used to perform an overall analysis which may lead to generating a stability index to use in identifying marginal behavior, monitoring slopes of post-licensure studies to identify potential shifts or supporting out-of-specification mitigation. Evaluating the impact of life-cycle management changes is another area where statistical stability models may be important tools post-licensure. Strategic uses of accelerated stability, as an extension of the development studies used to support vaccine licensure, can establish stability comparability after a process or manufacturing site change.

Dr. Andrew Lennard, Amgen, addressed alternative approaches for assessing the stability of mAb-based therapeutics and vaccines that support accelerated development, in contrast to conventional stability studies. Risk-based predictive stability uses principles of Quality by Design (QbD) to justify moving elements of conventional stability studies to post-approval by including the use of appropriate models to predict the product stability profile and establishment of shelf-life. Such predictive modeling approaches generally provide earlier assurance that these new vaccines will have the product quality and stability to meet the medical need during the designated shelf-life and administration to the patients at the vaccination site.

Two approaches were described in detail – prior knowledge stability models (such as using reference data sets for “like molecules”) and advanced kinetic analysis (such as using multiple accelerated temperatures to develop kinetic models) to predict shelf-life beyond the product-specific real-time data.

**Fig. 4:**
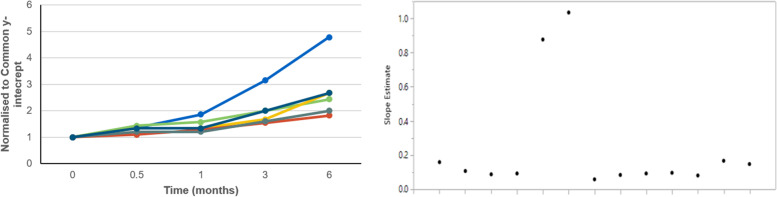
Kinetics of Impurity Formation under Accelerated Conditions. Excerpt from Lennard, A., Taking Stability Off the Critical Path of Vaccine Product Development, AAPS Vaccine Stability Workshop, March 24-25, 2021

The first approach leverages prior stability knowledge from platform product development (European Medicines Agency (EMA), 2017; European Medicines Agency/Food and Drug Administration (EMA/FDA), 2018; Quality by Design—An Indispensable Approach to Accelerate Biopharmaceutical Product Development, 2021). This approach relies on reference data sets for “like molecules”. To demonstrate transferable prior knowledge, the molecules selected for the Reference Data Set must meet pre-determined criteria. Product criteria for inclusion in the reference set and application of the model may be based on the modality, formulation, manufacture, container closure, storage conditions, and test methods. Stability data from accelerated conditions is a valuable tool in assimilating the reference data set, evaluating the fit of a new investigated product to the model, or identifying a non-fit molecule (Fig. 4, molecule 3, blue line on left plot, lots 3a, 3b on right plot). Such prior stability knowledge concept has been successfully applied to the shelf-life prediction of one of the COVID-19 vaccines (Part II) developed and manufactured using a platform technology The approach was acceptable to the regulatory agency for approval of EUA.

The second approach fits the best kinetic function to attribute stability data obtained at several temperatures that are then used to extrapolate the recommended storage temperature stability data to determine shelf-life. Although biologic/vaccine stability attributes may have complex kinetics, some key stability attributes can follow predictable kinetics within the necessary temperature ranges to allow kinetic models to be developed for stability and shelf-life purposes. Such kinetic modeling does not require an underlying mechanistic basis. An example was provided where an accurate stability prediction was achieved with 20 data points over 4 temperatures (Clenet, 2014).

The selection of the appropriate stability modeling approach may depend on the specific vaccine. Prior knowledge approaches require sufficiently similar approved products, with an understanding of the primary degradation pathways and key shared stability-indicating critical quality attribute (CQA). Kinetic modeling requires predictable product degradation kinetics, at least over the temperature ranges of interest. Both approaches can be used independently or complementarily to continuously support the determination of shelf-life as data accrue for the new product. The approaches discussed in both presentations bring a scientific focus that goes beyond traditional long-term stability and can increase both speeds of development and depth of understanding to the stability program.

### Part IV – Challenges of Vaccine Distribution and Deployment

**Fig. 5:**
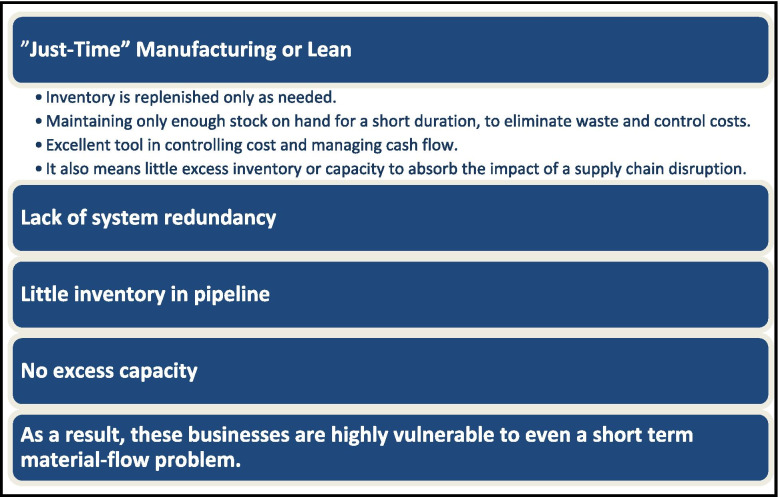
Reliance on Lean Concept for Supply Chain Management. Excerpt from Sanders, N., Challenges of Vaccine Distribution and Deployment, AAPS Vaccine Stability Workshop, March 24-25, 2021

In the last session, there were two speakers presenting topics about supply chain, logistics, and distribution and deployment of vaccines. Dr. Nada Sanders, from Northeastern University, gave a talk on the ‘Challenges of Vaccine Distribution and Deployment’. The talk educated the audience about different elements of the global supply chains and the challenges we have faced and/or are facing during the COVID-19 pandemic. Dr. Sanders introduced the interdisciplinary nature and critical traits, including managing bottlenecks and inventories. She addressed how the pandemic made global supply chains so vulnerable to disruption, including the complexity of modern supply chain, poor visibility, heavy reliance on lean and just-in-time processes, scaling/managing capacity, special requirements (e.g., cold chain), and the fact that productions can be concentrated in offshoring geographic areas (Fig. 5). She discussed remediation strategies for rebuilding resilience into supply chains. These include moving towards a digital supply chain, better relationships with suppliers, as well as strategic management of inventories.

To conclude the workshop, Dr. Tony Reed, Temple Health Hospital, presented the challenge of ensuring proper storage and handling at the vaccination center during the last mile. Taking the activities at Temple Health as an example, he described how the COVID-19 mRNA vaccines introduced unique challenges to the health care environment and unparalleled stress on our general logistics infrastructure. The challenges include receipt and storage of the vaccines that require -80°C storage condition, allocation, and distribution to multi-state residents with different priorities, selecting receivers, and coordinating schedules. These complexities increased with the need for two doses, holidays, weather, etc.

It is fascinating to learn of the challenges in solving for transportation and delivery at sub-arctic temperatures in a situation where the two available mRNA vaccines did not have the same storage temperature or allowed time for excursions. These vaccines were administered with two doses of the same type. At the time, they were not ‘interchangeable,’ thus, it was a challenge to assure that the right medicine was stored at the correct temperature at the right location to be administered at the right time. Temple Health System continuously improved its operation to ensure that the vaccine was delivered accurately to those in need. Dr. Reed presented an example spreadsheet to show the complexity of the coordination and how they used math and data analysis science to solve the challenges. Despite many obstacles, it was truly amazing that in early 2020 the healthcare industry learned to balance ‘just in time’ and advanced planning supply chains for acquisitions of medical supplies such as personal protective equipment, and laboratory testing reagents. By the end of 2020, their team had learned the science and art of cold-chain logistics in an end-to-end pathway.

The extremely complex logistics set up by the team was admirable from a systems organization perspective. Equally striking was the complex and constant training required for the personnel who were responsible for various steps during administration. This presentation reminded the audience about the importance of designing a robust vaccine delivery program even in the presence of human errors. It also highlighted the importance of establishing and harmonizing requirements for in-use and freeze-thaw stability studies, which are typically conducted at a later phase, to support product transport and storage in the distribution center. Since the vaccines are stored at such low temperatures, data from in-use and freeze-thaw stability studies can be very critical in developing a distribution plan.

## Conclusion

The two-day workshop on “Vaccine Stability Considerations to Enable Rapid Development and Deployment”, consisted of seven presentations, four-panel discussions, and 12 speakers, gathering almost 70 participants from many different pharmaceutical and healthcare companies. Among the recurring strategies discussed by multiple presenters and panelists throughout the workshop were: leveraging prior knowledge from existing vaccines, using kinetic modeling with accelerated stability data, and understanding the supply chain to ensure the success of the vaccine program. Without the use of these tools, it would not have been possible to receive EUA for two mRNA COVID-19 vaccines from the FDA in as little as 10-months from the start of the pandemic. The new knowledge gained from the development of the COVID-19 vaccine will likely shift the vaccine development, or even entire drug development into a new paradigm where platform development is used, and scientific modeling is applied. We are hopeful that any additional knowledge gained during the current COVID-19 pandemic will be useful in the fight against future pandemics in the years to come.

## Data Availability

Information in this article is summaries of the materials listed in the references or presented at the AAPS virtual workshop on “Vaccine Stability Considerations to Enable Rapid Development and Deployment,” on March 24-25, 2021.

## Abbreviations

AAPS: American Association of Pharmaceutical Scientists
TPP: Target Product Profile
EUA: Emergency Use Authorization
HTF: High Throughput Fluorescence
DSF: Differential Scanning Fluorimetry
GMP: Good Manufacturing Practices
BARDA: Biomedical Advanced Research and Development Authority
NIH: National Institutes of Health
FDA: Food and Drug Administration
DP: Drug Product
PCR: Polymerase Chain Reaction
SDS-PAGE: SDS-polyacrylamide gel electrophoresis
RP-HPLC: Reserved Phase High-Performance Liquid Chromatography
MALS: Multi-Angle Light Scattering
LC-MS: Liquid Chromatography-Mass Spectrometry
CMC: Chemistry and Manufacturing Controls
WHO: World Health Organization
LCM: Lifecycle Management
QbD: Quality By Design
CQA: Critical Quality Attribute
